# „Ein Social-Media-Post ist kein Projektil“ – Konzeptionelle Herausforderungen durch Desinformation

**DOI:** 10.1007/s12399-023-00937-9

**Published:** 2023-02-02

**Authors:** Adrian Teetz

**Affiliations:** Bundesministerium der Verteidigung, Stauffenbergstraße 18, 10785 Berlin, Deutschland

**Keywords:** Hybride Bedrohungen, Desinformation, Öffentliche Debatte, Informationskrieg, Narrative, Hybrid threats, Disinformation, Public debate, Information warfare, Narratives

## Abstract

Das sicherheitspolitische Paradigma der „Hybriden Bedrohungen“ zählt Desinformationsaktivitäten zum Arsenal internationaler „Grauzonenkonflikte“ unterhalb der Schwelle des bewaffneten Angriffs. Demokratische Staaten geraten damit in das Paradox, ihre Öffentlichkeit als Plattform der Meinungs- und Willensbildung gegen feindselige Interventionen zu verteidigen, ohne in deren konstitutive Freiheitlichkeit einzugreifen. Dies stellt die staatliche Gefahrenabwehr vor neue konzeptionelle Herausforderungen.

## Abstract (en)

The security policy paradigm of “hybrid threats” counts disinformation activities towards the different weapons of international “grey zone conflicts”, positioning them below the threshold of armed attacks. Democratic states are thus caught in the paradox of defending the public sphere – the ground upon which will and opinion form and develop – against hostile interventions without interfering in constitutive freedoms. This poses new conceptual challenges for state security.

## Einleitung

Die Öffentlichkeit in demokratischen Staaten wird durch Grundrechte geschützt, sie unterliegt kaum Regelungen. Ihre Funktionalität als Plattform pluralistischer Debatten zur politischen Meinungs- und Willensbildung stützt sich stark auf Konventionen, also freiwillig auferlegte Verhaltensmaßregeln der Beteiligten. Konventionen sind indes fragil. Angenommen, eine Person der Zeitgeschichte wird in der Öffentlichkeit mit einer herabwürdigenden Geste belegt. Falls sie dies im Affekt gleichermaßen erwidert, hat sie selbst öffentlich für sich die Konvention aufgehoben, nach der diese Geste verpönt ist. Zum Schutz einer öffentlichen Debatte gehört also auch, mit gezielten Provokationen durch Konventionsbrüche umzugehen.

Diese demokratische Öffentlichkeit gerät seit einigen Jahren in das Blickfeld der Sicherheitspolitik. Die Nationen der NATO und der EU sehen sich „Hybriden Bedrohungen“ ausgesetzt, zu denen auch Desinformationsaktivitäten gerechnet werden. Die Aufgabe, geeignete Maßnahmen zur Abwehr möglicher Risiken durch Desinformationen abzuleiten, erzeugt einige konzeptionelle Herausforderungen.

Neben der Klärung, welches Mandat die Regierung in diesem Kontext gegenüber der Öffentlichkeit ausübt, gehört auch dazu, die Implikationen für die Freiheitlichkeit und die Konventionen der öffentlichen Debatte besonders zu berücksichtigen. Der folgende Beitrag referiert zunächst aus einer Praxisperspektive^1^ exemplarisch den Stand der einschlägigen sicherheitspolitischen Debatte, ihr zugrundeliegender Annahmen und bisheriger Maßnahmen. Danach werden, insbesondere hinsichtlich der Vergleichbarkeit mit konventioneller Kriegführung, Schlussfolgerungen gezogen und weiterführender Forschungsbedarf skizziert.

## Hintergrund: Desinformation als „Hybride Bedrohung“

Bei der völkerrechtswidrigen Annexion der Krim 2014 hatte Russland, anders als im Februar 2022, die Ukraine noch nicht offen militärisch angegriffen. Stattdessen wurden irreguläre Kräfte eingesetzt, Separatisten militarisiert und die Landnahme mit systematischen Desinformationskampagnen vernebelt. Dieser auf europäischem Boden außergewöhnliche Gewaltakt löste in der NATO einen sicherheitspolitischen „Paradigmenwechsel“ (Schmid 2021, S. 221) mit erhöhter Aufmerksamkeit für sogenannte „Hybride Bedrohungen“ aus.

Grundsätzlich beschreibt diese Begrifflichkeit, dass ein Aggressor oder eine Konfliktpartei ein Spektrum unterschiedlicher Mittel von Politik über Militär, Wirtschaft, Finanzen, Recht bis hin zu Nachrichtendiensten (zunächst) unterhalb der Schwelle eines bewaffneten Angriffs planvoll einsetzt (Schaurer und Ruff-Stahl 2016, S. 9). Auf diese Weise entstehe ein permanenter „Grauzonenkonflikt“ im kontinuierlichen „Wettbewerb“ von Großmächten bzw. zwischen z. B. demokratischen und autoritären Systemen (vgl. Jordan 2020).^2^

Im Verhältnis zu ursprünglichen Konzepten aus den USA, die Gesichtspunkte wie irreguläre Kräfte und Terrorismus in den Mittelpunkt stellten, betont die deutsche Lesart seit 2014 „verstärkt die Propaganda, die Zerstörung Kritischer Infrastrukturen sowie die Handlungen im sogenannten Informationsraum“ (Kraft 2018, S 314). Damit rückten „Faktoren wie u. a. die psychologisch-moralische Beeinflussung des gegnerischen Willens oder der politisch-propagandistische Kampf um Informationshoheit und Legitimität als Gravitationszentren der Entscheidung in den Mittelpunkt“ (Schmid 2021, S. 229). Dieser spezifisch deutschen Perspektive im internationalen Kontext widmet sich der Schwerpunkt dieses Beitrages.

So ordnet das federführende Bundesministerium des Innern Desinformationskampagnen sprachlich als „Angriffe“ ein. Das gegnerische Vorgehen bestehe darin, „Nachrichten zu manipulieren oder aus dem Kontext zu reißen, um öffentliche Debatten zu beeinflussen“ (BMI 2022). Eine Bedrohung der nationalen Sicherheit erkennt das BMI demnach in der Absicht, „das Vertrauen in staatliche Stellen zu untergraben und durch das Befeuern kontroverser Themen gesellschaftliche Konflikte zu entfachen oder zu vertiefen“ (BMI 2022).

Dieses Bild entspricht auch dem „Aktionsplan gegen Desinformation“ der Europäischen Union (EK 2018, S. 3). Er definiert Desinformationen als „nachweislich falsche oder irreführende Informationen“ mit dem Ziel einer „vorsätzlichen Täuschung der Öffentlichkeit“. Sie haben zugleich das Potenzial, „öffentlichen Schaden“ anzurichten, worunter die EU „Bedrohungen für die demokratischen Prozesse sowie für öffentliche Güter wie die Gesundheit der Unionsbürgerinnen und -bürger, die Umwelt und die Sicherheit“ versteht (EK 2018, S. 1). Deutschland war nach Angaben einer EU-Agentur im Zeitraum zwischen 2015 und 2021 im europäischen Vergleich mit Abstand von den meisten russischen Desinformationsaktivitäten betroffen und steht demnach im Zentrum einer systematischen Kampagne (EUvsDisinfo 2021).

Als Reaktion hat die EU und in deren Rahmen Deutschland Maßnahmen zur Steigerung der gesellschaftlichen Resilienz und einer rechtlichen Regulierung von Social-Media-Plattformen ergriffen. Für Sicherheitsbehörden steht im Vordergrund, relevante Desinformationsaktivitäten zunächst einmal erkennen und in angemessener Zeit darauf reagieren zu können (EK 2018, S. 6–7; Rühle und Roberts 2021; Wigell et al. 2021, S. 8–9). Zugleich geht es darum, die politische Sichtweise der EU und der NATO-Staaten möglichst aktiv in der Öffentlichkeit bekannt und transparent zu machen sowie Desinformationen faktenbasiert entgegenzutreten und entsprechende zivilgesellschaftliche Initiativen zu unterstützen (EK 2018, S. 8–13).

Die NATO macht zudem einen ganzheitlichen Ansatz der Strategischen Kommunikation (NATO StratCom) zum integrierten Bestandteil ihrer politischen und Militärstrategie, was den koordinierten Gebrauch der Instrumente Public Diplomacy, (Military) Public Affairs, Information Operations und Psychological Operations einschließt (SCCOE 2020). Zu welchen Zwecken StratCom eingesetzt werden kann, stößt allerdings in NATO-Nationen auf unterschiedliche Auffassungen (s. u.).

## Desinformationen: Annahmen über Wirkungsmechanismen

Wie lassen sich die Risiken, die durch „Angriffe“ mit Desinformationsaktivitäten entstehen können, für die staatliche Gefahrenabwehr greifbar machen? In wissenschaftlichen und Praxisbeiträgen zur sicherheitspolitischen Debatte zeichnen sich wiederkehrende Annahmen über Wirkungsmechanismen ab, von denen einige Topoi hier überblicksartig zusammengestellt werden.

Ein sicherheitspolitisches Modell skizziert eine „strategische Logik“, die darauf zielt, gleichzeitig das Vertrauen einer Bevölkerung in die Demokratie sowie deren Institutionen zu untergraben und die „Anfälligkeit für polarisierende Narrative“ zu erhöhen (Ingram 2020, S. 17). Dies korrespondiert mit der Sorge vor einer „Spaltung“ der Gesellschaft bzw. einer Gruppierung der Öffentlichkeit zu „Echokammern“ und „Filterblasen“ durch algorithmisch auf individuelle Interessen zugeschnittene Oberflächen sozialer Medien (Jaursch und Sängerlaub 2020, S. 35–37; Nyhan 2020, S. 228; vgl. div. Beiträge in Zowislo-Grünewald und Wörmer 2021).

Den bereits erwähnten Narrativen wird dabei in verschiedenen Darstellungen eine zentrale Funktion zugewiesen. Ein Beitrag zu einer einschlägigen Fachtagung einer parteinahen Stiftung in Deutschland beschreibt „narrative Bedrohungen“ als drastisches Szenario: „Feindliche Narrationen unterminieren die Glaubwürdigkeit von Unternehmen, politischen Organisationen und im Extremfall der Demokratie als Staatsform.“ Wer dem nichts entgegenzusetzen habe, sei deren „Wirkung“ ausgeliefert: „Die Sicherheit der eigenen Überzeugungen bröckelt, möglich scheint alles bis zur Implosion des politischen Systems.“ (Zowislo-Grünewald und Hajduk 2021, S. 77).

Andere Beiträge des Tagungsbandes umreißen die angenommene Funktionsweise. So könnten durch „die Steuerung von Diskussionen in sozialen Netzwerken oder der [sic!] Platzierung von manipulativen Informationen auf Nachrichtenseiten“ potenziell „‚per Knopfdruck‘ ganze Bevölkerungsteile getäuscht, gespalten, polarisiert, abgelenkt oder im Sinne der eigenen Ziele mobilisiert werden.“ (Gruhl et al. 2021, S. 21) Narrative seien hierbei eine „Linse, durch die Einzelne die Informationen interpretieren und damit interagieren, und bestimmen die Art der moralischen Lehren und Schlussfolgerungen, die daraus gezogen werden.“ (Knappenschneider und Feige 2021, S. 194).

Offene, demokratische Gesellschaften werden hierfür vom BMI und anderen Autor*innen als „anfällig“ eingestuft, weil sie viele „Angriffsflächen für offene und verdeckte Aktivitäten“ böten (BMI 2022). Dass russische Staatsvertreter „es in die Schlagzeilen der großen Mainstream- und Qualitätsmedien“ schafften, wird dabei als besonderer Erfolg russischer Desinformation bewertet (Meister 2022), in einem NATO-Review ist von „vulnerable audiences“ die Rede (Rühle und Roberts 2021).

Die Terminologie der Debatte ist von militarisierten Metaphern durchzogen. Eine Analyse einschlägiger Dokumente konstatiert „information war“ als spezifisch russische Variante der „strategic communications“ (Fridman 2020, S. 46). Die Wirkung von Desinformationen wird metaphorisch mit der von Waffen verglichen, eine Autorin greift sogar zu der Formulierung, Russland habe soziale Medienplattformen „in psychologische Massenvernichtungswaffen verwandelt“ (Aro 2022, S. 42). Die Begrifflichkeiten suggerieren einen eigenständigen Konfliktschauplatz „Informationskrieg“, auf dem Informationen wie Waffen eingesetzt werden und ein „Kampf um Narrative“^3^ oder „Kampf um die Köpfe“ (Reimann 2022) herrscht.

Der damalige Director of Public Affairs^4^ der Canadian Armed Forces erwartete 2019, “that decisive narrative battles will take place primarily at the strategic level” (Janzen 2019, S. 4) und erörterte, die Funktion der Public Affairs, die traditionell auf eine „Information“ der Öffentlichkeit zielt, auch zu deren „Beeinflussung“ einzusetzen. Hierzu skizzierte er für die kanadischen Streitkräfte ein Konzept der “Ethical Influence”: “To be permissible, domestic PA influence efforts should be required to meet four key criteria: they must be truthful, transparent, helpful, and limited.” (Janzen 2019, S. 9–10).

Auch in dem bereits erwähnten Tagungsband schlägt ein Autorenduo „Counter-Influence-Kampagnen“ zur „Wiedererlangung der Deutungshoheit in der Online-Sphäre“ vor (Knappenschneider und Feige 2021, S. 198–199). Dazu müssten mit Hilfe von Open-Source-Intelligence-Technologien (OSINT) bereits in „halbgeschlossenen Räumen“ Desinformationsnarrative identifiziert und die Gruppendynamik ihrer Verbreitung analysiert werden (Knappenschneider und Feige 2021, S. 199–200). Auf dieser Grundlage seien geeignete Gegenkampagnen durch „Verbreitung von faktengeprüften Botschaften“ zu entwickeln (Knappenschneider und Feige 2021, S. 201).

## Herausforderungen: Lagebild und Beurteilung

Der bislang skizzierte Umriss der Debatte über Desinformationsaktivitäten als Bestandteil Hybrider Bedrohungen stellt Sicherheitsbehörden vor einige konzeptionelle Herausforderungen. Im Mittelpunkt steht dabei das Lagebild als Fundament politischer und administrativer Entscheidungen.

Dem Konzept der Hybriden Bedrohungen wohnt eine „beabsichtigte Diffusion der Form internationaler Konfliktaustragung“ (Schaurer und Ruff-Stahl 206, S. 9) inne, es bleibt amorph und eröffnet weite Spielräume der Auslegung. Da im digitalen Raum die Interventionsmöglichkeiten keine territorialen Grenzen kennen, stehen aus gesamtstaatlicher Perspektive traditionelle Aufgreifschwellen und Zuständigkeitsabgrenzungen einschließlich der daran geknüpften Legitimationsprozesse zur Diskussion. Ein bewaffneter Angriff, bei dem wie im Februar 2022 russische Truppen die territoriale Grenze der Ukraine überschreiten, ist offensichtlich – woran aber macht eine Exekutive den Fall eines „information war“ fest und wer entscheidet über dessen Eintritt?^5^

Die Debatte wird engagiert entlang prägnanter Metaphern geführt, deren Bedeutungsinhalte jedoch nicht allgemeinverbindlich definiert und interessengeleitet auslegbar sind. Eine begriffshistorische Analyse der Hybriden Bedrohungen reflektiert die Möglichkeit eines „Mythos“, mit dem sicherheitspolitische Akteure politische Aufmerksamkeit generieren und eine Ausweitung eigener Kompetenzbereiche begründen wollten (Kraft 2018, S. 316–318).

Auch die Kategorie „Desinformation“ bleibt für die Erstellung eines Lagebildes vage. Eine übergreifend etablierte Begriffsdefinition besteht nicht, unterschiedliche Vokabeln (etwa „Des-“, „Mis-“, „Malinformation“) werden zum Teil synonym verwendet (Jaursch und Sängerlaub 2020, S. 32–33). Die Bundesregierung differenziert zwischen „Fehl-“ (z. B. Irrtümer, „Zeitungsenten“) und „Desinformation“, wobei Letztere anhand einer „Täuschungsabsicht“ identifiziert wird (Bundesregierung 2022). Allerdings sind Urheber und Absichten von Desinformationen in aller Regel nicht offensichtlich und können nur attribuiert werden (Bayer 2020, S. 43–44). Inwieweit „manipulierte“ oder „aus dem Kontext gerissene“ (BMI 2022) Informationen tatsächlich gegnerischen Aktivitäten zugeordnet werden können, ist schwierig abzugrenzen bei realistischer Betrachtung einer freien öffentlichen Debatte, in der „false information, misperceptions, and conspiracy theories“ als „general features of human society“ (Nyhan 2020, S. 232) vorauszusetzen sind.

Auch die Tragweite einer Desinformationsaktivität ist im Vergleich zu einer Cyberattacke oder gar einem bewaffneten Angriff schwieriger einzuschätzen. Der hierbei zentral verwendete Begriff „Einflussnahme“ bleibt unbestimmt, er lässt Art, Richtung und vor allem Gewicht des Einflusses offen. Die drastisch anmutenden Zahlen der EU-Agentur (s. o.) werden im umgangssprachlichen Alltag oft als „Wirkung“ wahrgenommen, gewähren aber tatsächlich zunächst nur Aufschluss über die Intensität der beobachteten Desinformationsaktivitäten. Die Erhebung einer tatsächlichen „Wirkung“, z. B. auf Wahrnehmung oder Einstellungen von Rezipient*innen, kann nur punktuell erfolgen und ist mit erheblichem Aufwand verbunden (s. u.).

Vor dem dargestellten Hintergrund lassen sich die oben referierten Grundannahmen aus der Praxis über Wirkungsweisen nicht ohne Weiteres zu einem empirisch fundierten^6^ Lagebild über den Zustand der Öffentlichkeit und den Einfluss von Desinformationsaktivitäten zusammensetzen. Dass sich mit sozialen Medien „auf Knopfdruck“ Teile der Bevölkerung „spalten“ lassen könnten (Gruhl et al. 2021) deckt sich nicht mit einschlägigen Ergebnissen sozialwissenschaftlicher Forschung. Im Gegenteil: Wissenschaftler*innen mahnen zur Vorsicht bei der Bewertung möglicher Wirkungen von Desinformationen auf Verhalten und Einstellungen von Menschen (Möller und Hameleers 2020, S. 12) und haben eine Typologie einschlägiger Fehlinterpretationen (Altay et al. 2021, S. 4) herausgearbeitet.

Ein aktuelles Beispiel mag die Herausforderung illustrieren: In Deutschland hat eine NGO Zustimmungswerte zu bestimmten Aussagen im Kontext des russischen Angriffs auf die Ukraine erhoben, die Analogien zu typischen Narrativen russischer Propagandaquellen („pro-russische Verschwörungserzählungen“) aufweisen. Obwohl die Urheber des entsprechenden Research Papers selbst darauf hinweisen, dass ihr „Stimmungsbild“ eine „Wirkungskraft von Desinformationskampagnen nicht messen könne“ (Lamberty et al. 2022, S. 8), wird in Teilen der medialen Berichterstattung ein ursächlicher Zusammenhang wahrgenommen oder suggeriert (MDR 2022; Zeit Online 2022; Sächsische Zeitung 2022; KSTA 2022)^7^.

Auch die angebliche „Anfälligkeit“ offener Gesellschaften ist fraglich: Warum sollte es als Erfolg der Desinformation gewertet werden, wenn auch die Stellungnahmen russischer Staatsvertreter in den unabhängigen Medien referiert werden (Meister 2022)? Ist es nicht gerade sinnfälliger Ausdruck des Unterschieds zur regulierten Öffentlichkeit des autoritären Systems, dass sich jedes Individuum im Kontext der pluralistischen Debatte demokratischer Staaten hierüber ein eigenes Urteil bilden kann?

Die Befürchtung einer „gespaltenen“ Gesellschaft (s. o.) hat sich – jedenfalls für Deutschland – bislang ebenso wenig bestätigt (Mau 2022, S. 17–18, vgl. u. a. Westheuser 2022 sowie im internationalen Vergleich Silver 2021), wie die befürchteten „Echokammern“ und „Filterblasen“ (Ross Arguedas et al. 2022, S. 29–30; Bundestag 2022, S. 15–19). In den seit den Präsidentschaftswahlen 2016 sensibilisierten USA konnte zwar eine starke Intensität russischer Desinformationsaktivitäten, nicht aber ein relevanter Einfluss auf Einstellungen und (Wahl‑)Verhalten der Bevölkerung nachgewiesen werden (vgl. Benkler 2018; Karpf 2019) Zudem hätten die Technologieplattformen aus wirtschaftlichen Gründen kein Interesse, vertiefende Untersuchungen hinsichtlich der Wirksamkeit mit Daten zu unterstützen: Käme dabei heraus „that campaigns on the platform had no meaningful impact, then the company’s core business model – selling targeted ads on the promise that they will sway voters and consumers – will be shown to be ineffective“ (Benkler 2019).

Einige Forschende sehen in den „alarmistischen Narrativen“ über Desinformationen den Ausdruck einer „Technopanik“ (Altay et al. 2021, S. 2) oder „Emotionalisierung bis hin zur Panikmache“ (Meier und Hoffmann 2022). Andere warnen davor, die öffentliche Wahrnehmung des Themas selbst übermäßig zu stimulieren: „Nonstop coverage of propaganda efforts and speculation about their impact, without actual evidence to support that impact, feeds the loss of trust in our institutions to a greater extent than the facts warrant“ (Benkler 2019). Permanente Thematisierung von Desinformationen trage zu Skeptizismus gegenüber Politik und Medien bei (Nyhan 2020, S. 233).

Dies führt zu einem weiteren Aspekt des Lagebildes, der Differenzierung von primären und sekundären Effekten: Zielte traditionelle Propaganda vor allem darauf ab, unmittelbar Meinungen und Einstellungen ihrer Zielgruppen zu beeinflussen (Bussemer 2008, S. 35–36), richten sich aktuelle Desinformationsaktivitäten wesentlich darauf, die Funktionalität öffentlicher Debatten demokratischer Staaten zu stören, abzulenken und sie insgesamt zu desintegrieren (Nordheim 2020; Karpf 2020, S. 161–165). Die Konventionen, auf denen Integrität und Funktionalität einer öffentlichen Debatte als konstitutivem Austragungsort politischer Verhandlungen in der Demokratie gründen, sind also ein kritischer Faktor der Lagebeurteilung.

In dieser Übersicht wird deutlich, vor welche konzeptionellen Herausforderungen der diffuse Charakter der Bedrohungslage Sicherheitsbehörden schon bei der Konfiguration eines Lagebildes stellt – auf dessen Grundlage wiederum das Geschehen erfasst, beurteilt und mögliche Maßnahmen abgewogen werden müssen. Verzerrungen im Lagebild wiederum können konsequente Fehlurteile und -entscheidungen nach sich ziehen. Eine Fokussierung auf Desinformationsaktivitäten könnte zudem dazu führen, dass andere Ursachen für z. B. tangiertes Institutionenvertrauen aus dem Blickfeld gerieten.

Warum die oben skizzierten Annahmen über Wirkungsmechanismen mit der Gegenüberstellung sozialwissenschaftlicher Forschungsergebnisse nicht kongruent sind, könnte hier nur spekuliert werden. Ein Deutungsansatz könnte darin liegen, dass die Aufarbeitung des Themas Desinformation Bestandteil der Debatte über Auswirkungen der Digitalisierung auf die Öffentlichkeit ist, die seit ca. zehn Jahren entlang griffiger Metaphern wie „Echokammer“ etc. geführt wird. Solcherart semantische Komplexitätsreduktion erleichtert „auf der einen Seite effiziente Kommunikation, hat aber auf der anderen Seite den Effekt, dass jene Begriffe und Konzepte durch die Diskursteilnehmer*innen bald selbst als Realität begriffen werden“ (Kraft 2018, S. 315). Die oben referierte Kriegsmetaphorik ist zudem ein Hinweis darauf, dass die Konfrontationen mit Desinformationen in Sicherheitsbehörden, insbesondere militärischen, auf traditionelle Annahmen über Ursache-Wirkungs-Beziehungen aus der konventionellen Kriegführung stößt.

## Mandate der Regierungskommunikation: Unterschiedliche Auffassungen in NATO-Nationen

An die Herausforderung einer validierten Lagebeurteilung schließt die Ableitung spezifischer Aufgaben staatlicher Gefahrenabwehr gegenüber Desinformationsaktivitäten an. Im Mittelpunkt steht die Frage, welches Mandat Regierungsstellen grundsätzlich gegenüber der inländischen Öffentlichkeit ausüben. Hierzu existieren unter den Mitgliedsnationen der NATO unterschiedliche Auffassungen, deren Spektrum sich an einer Gegenüberstellung der Positionen aus Großbritannien und Deutschland aufzeigen lässt.

Die britische Regierung hat seit 2010 die Methoden und Ziele ihrer Regierungskommunikation auf Grundlage eines verhaltensökonomischen^8^ Konzepts (Graf 2018, S. 436). grundlegend neu ausgerichtet: „Communications is not simply broadcasting the work of government after the fact. We are one of the vital levers government uses to realise its objectives and implement public policy. The success of that policy inevitably involves people starting, stopping or changing behaviours“ (UK Government 2018, S. 5). Hierzu stellen die Regierungsbehörden zyklisch Kampagnenpläne mit Kommunikationszielen auf, die messbar definiert sein sollen. Auf der Ebene der Ausführungsdokumente wird der Anwendungsbereich dieser Ziele auf Einstellungen der Bevölkerung erweitert: „Objectives should be […] related to changing attitudes and/or behaviour“ (UK Government 2020).^9^ Für Zwecke der nationalen Verteidigung soll der verhaltensorientierte Ansatz ebenfalls angewendet werden, und zwar ausdrücklich für alle Zielgruppen, „not only hostile parties (‘the enemy’) but also opposing, neutral, supportive and friendly audiences“ (UK MoD 2019, S. 24).

Dem lässt sich das Verständnis von Regierungskommunikation in Deutschland gegenüberstellen. So beruft sich z. B. das Bundespresseamt bei der Darstellung seiner Aufgaben auf einschlägige Rechtsprechung des Bundesverfassungsgerichts: Die Regierung „muss die Bürgerinnen und Bürger über entscheidende Sachfragen umfassend informieren. Nur so kann jede Einzelne und jeder Einzelne die getroffenen Entscheidungen, Maßnahmen und Lösungsvorschläge richtig beurteilen, sie billigen oder verwerfen“ (BPA 2022). Mit kommunikativen Maßnahmen soll die Regierung eine „verantwortliche Teilhabe“ der Bürgerinnen und Bürger unterstützen. Dies sei ihnen nur möglich, „wenn die staatliche Informationstätigkeit so gestaltet ist, dass sie einen reflektierten und rationalen Umgang mit sich erlaubt.“ (Mast 2020, S. 61) Informationsstrategien, „die einen reflektierten Umgang mit sich gerade unterbinden, ihre wahre Intention verschleiern und stattdessen auf manipulative Weise wirken wollen“, seien demgegenüber illegitim (Mast 2020, S. 61). „Informieren“ hat im Sinne des deutschen Grundgesetzes also normativen Charakter, das auf einem positiven „Menschenbild“ (Teetz 2019, S. 92–98) im Respekt vor der Selbstbestimmung des Individuums gründet.

Diese Gegenüberstellung veranschaulicht das Spektrum unterschiedlicher Auffassungen innerhalb der NATO-Nationen, welche Befugnisse einer Regierung gegenüber der Öffentlichkeit zugemessen werden. Während in Großbritannien der Einsatz staatlicher Kommunikationsressourcen einer temporär gewählten Regierung zur Beeinflussung des Souveräns offensichtlich unproblematisch erscheint, träfe dies in Deutschland auf grundlegende Restriktionen der Verfassung (Fuhrberg 2020, S. 93–96).

## Risiken: Überreaktion und unbeabsichtigte Effekte

Wie dargelegt, deklarieren die zitierten Auslegungen im Rahmen des Konzepts der Hybriden Bedrohungen das (territorial) grenzenlose „digitale Informationsumfeld“ zu einem sicherheitspolitischen Schauplatz. Daraus folgt, dass die Öffentlichkeit in ihrer Eigenschaft als Plattform pluralistischer Debatten zur Meinungs- und Willensbildung als Objekt staatlicher Gefahrenabwehr betrachtet wird. Eben diese Öffentlichkeit genießt auf Grund ihrer konstitutiven Bedeutung für die demokratische Staatsform allerdings den ausdrücklichen Schutz des Grundgesetzes – auch vor staatlicher Intervention. Das ist zugleich ein herausragendes Unterscheidungsmerkmal gegenüber jenen autoritären Systemen, die das sicherheitspolitische Paradigma des „continuum of competition“ (s. o.) als permanente Gegner ausmacht.

Vor dem Hintergrund dieses Spannungsfeldes erzeugen Desinformationsaktivitäten das immanente Risiko einer Überreaktion oder Reaktion mit untauglichen Mitteln und unbeabsichtigten Effekten. Beispielhaft lassen sich solche Risiken an der Vokabel „Informationskrieg“ festmachen, die in der Debatte oszilliert. Der Begriff suggeriert, wie erwähnt, einen quasi ballistischen Schlagabtausch mit Informationen, die wie Waffen eingesetzt würden. Tatsächlich ist der Begriff jedoch in der Terminologie der NATO nicht definiert.

Wenn „information war“, wie es die zitierte begriffshistorische Analyse (Fridman 2020, S. 46) nahelegt, tatsächlich die russische Entsprechung zum Konzept der „strategic communication“ wäre – sind demokratische Staaten dann gut beraten, die Terminologie und die daran geknüpften Implikationen von ihren Gegnern zu übernehmen? Sollen sie sich – ungeachtet der Frage, ob das so überhaupt funktioniert – ernsthaft selbst mit „Informationswaffen“ verteidigen, indem sie auf Öffentlichkeiten „einwirken“^10^? Selbst wenn im Inland eine relevante Beeinflussung durch Desinformationsaktivitäten nachgewiesen werden könnte – was bislang nicht gegeben ist (s. o.) – hätten demokratische Regierungen nur sehr bedingt ein Mandat, mit staatlichen Kommunikationsinstrumenten „Counter-Influence“ auf ihren Souverän, die Bevölkerung, auszuüben.

Die suggestive Ausrufung eines „Informationskrieges“ durch russische Staatsmedien (Nimmo 2018) sollte demokratische Staaten – wie in der Einleitung am Beispiel angemerkt – nicht dazu provozieren, sich mit Mitteln zu „verteidigen“, die in Widerspruch zu den eigenen Werten und Normen geraten. Eindringlich beschrieben hat dieses Risiko die US-amerikanische Expertin Nina Jankowicz^11^: „But establishing a coherent counter-disinformation policy becomes impossible, when governments stealthily crack open the Russian playbook for political gain; the line between fact and fiction is blurred even further and levels of distrust grow when governments engage in the same technique as Russia, becoming purveyors of disinformation themselves“ (Jankowicz 2020, S. 203).

Dies korrespondiert mit der Befürchtung von Wissenschaftler*innen, politische Eliten könnten angesichts der Allgegenwart von Desinformationen den Respekt vor der intellektuellen Selbstbestimmung ihrer Bürger*innen sowie den Kontrollmechanismen der demokratischen Öffentlichkeit („the myth oft the attentive public“) verlieren und sich daher an Debattenkonventionen nicht mehr gebunden fühlen (Karpf 2019). Diverse Veröffentlichungen heben die Bedeutung des Verhaltens von Eliten als Rollenvorbilder für die Funktionalität der öffentlichen Debatte hervor (Nyhan 2020, S. 227; Ross Arguedas et al. 2022, S. 25–28).

Hinzu kommen Unsicherheiten über die Eignung des Instrumentariums an Vorsorge- und Reaktionsmöglichkeiten. So, wie eine Wirkung von Desinformationen nur bedingt vorausberechnet werden kann, gilt dies auch für eventuelle Gegenmaßnahmen. Es ist immer im Einzelnen abzuwägen, ob eine Reaktion per se zur Weiterverbreitung problematischer Botschaften, zur Reaktanz (vgl. Steindl et al. 2015) oder zur Nachrichtenvermeidung (Hölig et al. 2022, S. 12–15) beitragen kann. Solche unbeabsichtigten Effekte wiederum könnten womöglich selbst die Funktionalität der öffentlichen Debatte tangieren und damit auf die sekundäre Zielsetzung von Desinformationsaktivitäten einzahlen. Dieses Risiko ist bei der Abwägung von Reaktionsmöglichkeiten gleichrangig mitzubetrachten.

Daran wird zugleich deutlich, wie problematisch der Vergleich von Informationen und Waffen ist: Im Gebrauch realer, kinetischer Waffen können Soldat*innen Flugbahn und Wirkung eines Projektils vergleichsweise exakt einschätzen. Ein Social-Media-Post aber ist kein Projektil und verhält sich in der Dynamik digitaler Massenkommunikation weit weniger vorhersehbar. So scheint auch ein einschlägiges Experiment der kanadischen Streitkräfte während der Covid-19-Pandemie jedenfalls nicht die intendierten Ergebnisse^12^ erzielt zu haben.

## Schlussfolgerungen

Die vorliegende Arbeit zeigt, welche konzeptionellen Herausforderungen die Konfrontation mit Desinformationsaktivitäten im Rahmen Hybrider Bedrohungen für Sicherheitsbehörden in demokratischen Staaten und insbesondere in Deutschland erzeugt.

Dabei wurden drei wesentliche Gesichtspunkte herausgearbeitet:

- Die Schwierigkeit, ein unverzerrtes Lagebild über Desinformationsaktivitäten zu erstellen, das eine Beurteilung ihrer Relevanz und Wirksamkeit und mithin die Abwägung von Reaktionsmöglichkeiten belastbar unterlegt. Unsicherheiten bestehen hier gleichermaßen gegenüber der Wirkung wahrgenommener gegnerischer Aktivitäten wie gegenüber möglichen eigenen Mitteln.
- Das Paradox, die Öffentlichkeit demokratischer Staaten gegen feindselige Interventionen zu verteidigen, ohne in deren konstitutive Freiheitlichkeit einzugreifen.
- Das Risiko, sich zu unangemessenen Reaktionen provozieren zu lassen und somit selbst zu einer Beschädigung der öffentlichen Debatte beizutragen.

Eine wesentliche Schlussfolgerung besteht darin, dass Annahmen über Ursache-Wirkungs-Beziehungen und Begrifflichkeiten aus der konventionellen Kriegführung nicht ohne Weiteres auf die Auseinandersetzung mit Desinformationsaktivitäten übertragbar sind. Eine solche Übertragbarkeit legt die sicherheitspolitisch zirkulierende Metaphorik eines „Informationskriegs“ und des „Kampfs um Narrative“ mit „Informationswaffen“ jedoch nahe. So prägnant und naheliegend die Metaphern sein mögen, sie erzeugen das Risiko, sich zu verselbstständigen und zu Fehlschlüssen einzuladen.

Statt sich hier womöglich das Vokabular des Gegners (vgl. Kap. 3 und Kap. 6) anzueignen, ist zunächst angezeigt, das Instrumentarium der Lagebeschreibung und -beurteilung zu fundamentieren. Gemeinsame Definitionen und Begriffe können weiter ausdifferenziert werden und zusammen mit werte- und normenbasierten Grundsätzen ein ressortübergreifendes Konzept formen, um sich in föderaler und subsidiärer Verantwortung kohärent mit Desinformationen auseinanderzusetzen. Eine angemessen schnelle und zielgerichtete Zusammenarbeit beteiligter Behörden lässt sich zunächst prozessual darstellen und durch Sensibilisierung des Personals bzw. Fortbildungsprogramme ergänzen. Über Bedarf für mögliche weitergehende Institutionalisierung des Aufgabengebietes lässt sich wiederum besser auf Grundlage eines belastbaren Lagebildes über Intensität und Tragweite möglicher Risiken urteilen.

Vor diesem Hintergrund ist ein weitergehendes Forschungsinteresse unter den folgenden Gesichtspunkten absehbar.

### Konsolidierung des Lagebildes.

Bislang sieht sich die Verwaltungspraxis mit einer großen und disparaten Summe partikularer Forschungsergebnisse konfrontiert, die sehr unterschiedliche Auslegungen nach sich ziehen können. Als systemischer Ansatz könnte z. B. der Bezugsrahmen für Kommunikationscontrolling der einschlägigen deutschen Berufsverbände^13^ herangezogen werden. Analog wirtschaftlicher Kennzahlensysteme skizziert das Modell insgesamt vier mögliche Prozessstufen kommunikativer Aktivitäten und deren Wirkung (Input, Output, Outcome, Outflow), berücksichtigt dabei aber die zahlreichen Variablen: „Gezielte Kommunikationsmaßnahmen führen jedoch keineswegs zwangsläufig zu Einstellungs- und Verhaltensänderungen bei den Rezipienten, da Kommunikationswirkungen stets von den Interessen aller Beteiligten beeinflusst werden. Der Einfluss externer Faktoren nimmt von Input zu Outflow zu, während der Einfluss des Kommunikationsmanagements zwangsläufig stufenweise abnimmt“ (Zerfass 2015, S. 7).

Zum einen unterlegt dieser Bezugsrahmen ein gemeinsames Verständnis kommunikativer Ursache-Wirkungs-Beziehungen und insbesondere der Definition von „Wirkung“, zum anderen können dabei unterschiedliche Wirkungsebenen wie z. B. Reichweite („Externer Output“) und Wahrnehmung („Direkter Outcome“) differenziert werden. Auf diesen beiden Wirkungsstufen ließen sich zugleich legitime Ziele der Regierungskommunikation abbilden und mit beherrschbarem Aufwand messen, z. B. Relevanz in der öffentlichen Debatte und eine sinngetreue Wahrnehmung des Sachverhalts und/oder der Quelle als glaubwürdig. Ob der Souverän sich von entsprechenden Aktivitäten beeindrucken und seine Einstellungen und sein Verhalten daraufhin ändert, bleibt ihm bei diesen Zielsetzungen a priori selbst überlassen. Allerdings wurde das Konzept für Zwecke der Wirtschaftskommunikation erarbeitet und müsste für die spezifischen (z. B. normativen) Voraussetzungen der Regierungskommunikation wahrscheinlich angepasst werden.

### Handlungsrahmen regierungsamtlicher Öffentlichkeitsarbeit unter digitalen Bedingungen in Deutschland.

Noch immer gründet die Praxis hier weitgehend auf der Auslegung von zurückliegender Verfassungsrechtsprechung aus teilweise vordigitaler Zeit, jedoch haben sich die Anforderungen an z. B. Responsivität (Dialogfähigkeit) öffentlicher Verwaltung verändert, die wiederum digital selbst über Möglichkeiten (potenzieller) Massenpublikation verfügt (vgl. Kocks et al. 2020). So liegt mit Mast (2020) bislang erst eine juristische Studie vor, die aktuelle sozialwissenschaftliche Erkenntnisse in die juristische Bewertung systematisch einbezieht und einen Rahmen für die Kommunikation der öffentlichen Hand skizziert. Im Übrigen bedarf es spezifischer Regelungen für den Fall der Landes- und Bündnisverteidigung.

## Footnotes

1 Aus der Perspektive von Sicherheitsbehörden gilt es, in einer dynamischen und teilweise widersprüchlichen Informationsumgebung für die Praxis verwertbare Schlussfolgerungen abzuleiten. Daher wurden hier Quellen interdisziplinär von unterschiedlicher, nicht nur wissenschaftlicher Herkunft herangezogen. Soweit zweckmäßig, werden die Quellen kontextualisiert.

2 Grundsätzlich gelten auch Spielarten des internationalen Terrorismus als Ausgangspunkt Hybrider Bedrohungen, dieser Beitrag fokussiert aber auf internationale Konflikte zwischen den Interessenssphären von Großmächten bzw. unterschiedlichen politischen Systemen. Die sicherheitspolitische Debatte um Hybride Bedrohungen verläuft dynamisch und mit wechselnden bzw. neu hinzukommenden Aspekten (vgl. Kraft 2018). Dieser Beitrag, der auf den Informationsraum und dessen Bedeutung für demokratische Normen und Werte abzielt, kann die Debatte nur selektiv wiedergeben.

3 Ein Briefing des EU-Parlaments von 2016 über NATO StratCom trägt die Überschrift „An evolving battle of narratives“ (Europäisches Parlament 2016). Bemerkenswerterweise kommt die Metapher „battle of narratives“ jedoch ausschließlich in der Überschrift, nicht aber im Text des Dokuments vor und wird weder eingeführt noch definiert.

4 Dieser ist seit Juni 2021 Director der Communications Division im militärischen NATO Hauptquartier SHAPE, Brüssel.

5 Das britische Verteidigungsministerium geht ausweislich eines Dokumentes zur „Strategic Command Strategy“ von folgender Situation aus: „The distinction between war and peace no longer applies“ (UK MoD 2021).

6 Die Landschaft relevanter Forschungsergebnisse ist interdisziplinär, umfänglich und zum Teil disparat, hier können nur beispielhafte Gesichtspunkte aufgegriffen werden ohne Anspruch auf Vollständigkeit. Dies veranschaulicht zugleich die Herausforderungen, vor denen Sicherheitsbehörden bereits bei der Erstellung eines Lagebildes stehen.

7 Beispielhaft veranschaulichen diesen Wahrnehmungseffekt die Überschriften der einschlägigen Berichte, in denen es heißt „Propaganda wirkt“ (MDR), Deutsche „stimmen russischer Propaganda zu“ (Zeit Online), würden von dieser „überzeugt“ (Sächsische Zeitung) oder „glaubten“ ihr (KSTA).

8 Die sog. „Verhaltensökonomik“ ist ein wirtschaftswissenschaftliches Konstrukt, das menschliches Verhalten vor dem Hintergrund von Ergebnissen psychologischer Forschung zu erklären und vorherzusagen versucht. Grundlage sind die Arbeiten des Psychologen Kahneman (2011), der Entscheidungsprozesse im menschlichen Gehirn vermessen hat und das darauf basierende Politikberatungskonzept von Thaler und Sunstein (2011). Bei Letzterem geht es im Kern darum, die Umgebung, in der Menschen Entscheidungen treffen, so auszugestalten, dass diese „richtig“ entscheiden („Libertärer Paternalismus“, Thaler und Sunstein 2011, S. 14–15). Kritische Reflexion findet sich methodisch bei Gigerenzer (2018), normativ bei Fuhrberg (2020).

9 Eine beispielhafte Zielformulierung aus dem „Government Communications Plan 2015/16“: „Securing real change in our relationship with Europe: The public wants to see a real change in our relationship with Europe. In the coming months, we will provide communications support to the renegotiation of our country’s ties with the EU to secure the best deal possible for the UK and plan for the EU Referendum by the end of 2017“ (UK Government 2015, S. 13). Eine solche Ausrichtung staatlicher Ressourcen der Regierungskommunikation auf die Unterstützung einer legislaturgebundenen Regierungsposition in einer staatspolitisch derart vitalen Frage stieße in Deutschland wahrscheinlich auf verfassungsrechtliche Bedenken.

10 Vgl. die aus dem Grundgesetz abgeleiteten „Gebote“ für Regierungskommunikation bei Mast (2020, S. 377–379) sowie die Reflexion über „mündige Bürger*innen“ und „Manipulation“ bei Fuhrberg (2020, S. 93–98).

11 Jankowicz war im Mai 2022 nur acht Wochen nach Amtsantritt als Direktorin des neu geschaffenen US Disinformation Governance Board zurückgetreten, nachdem es zu öffentlichen Kontroversen sowohl um die Einrichtung als auch ihre Person gekommen war (Bond 2022). Die neugegründete Behörde wurde im August des gleichen Jahres wieder aufgelöst (DHS 2022). Dieses Beispiel veranschaulicht die außerordentliche Sensibilität im Verhältnis von demokratischer Öffentlichkeit und staatlichen Interventionen, das allerdings auch vor dem Hintergrund der stärker polarisierten politischen Landschaft in den USA betrachtet werden muss (vgl. Silver 2021). Auch eine Initiative der französischen Regierung, während der Covid-19-Pandemie eine Positivliste mit vertrauenswürdigen Medien („greenflagging“) zu etablieren, wurde nach öffentlichen Protesten schnell wieder eingestellt (Artelt 2020).

12 Laut Medienberichten sollen die Canadian Armed Forces 2020 Techniken zur Beeinflussung der inländischen Öffentlichkeit (vgl. Janzen 2019) erprobt haben, was jedoch eher gegenteilige Reaktionen (Lamoureux 2021), sowie über einen längeren Zeitraum öffentliche Kontroversen und interne Untersuchungen nach sich gezogen habe (Pugliese 2021).

13 Dies sind z. B. die Deutsche Public Relations Gesellschaft (DPRG) und die deutsche Sektion des Internationalen Controller Vereins (ICV).

## References

[CR1] Altay S, Berriche M, Acerbi A (2021). Misinformation on Misinformation: Conceptual and Methodological Challenges.

[CR2] Aro J (2022). Desinformation als Waffe. Über einen Krieg, den Russland seit Jahren führt. Aus Politik und Zeitgeschichte.

[CR3] Artelt (2020, 16. Mai). Fact-Checking in Frankreich. „So funktioniert das nicht in einer Demokratie“. Deutschlandfunk Kultur. https://www.deutschlandfunkkultur.de/fact-checking-in-frankreich-so-funktioniert-das-nicht-in-100.html. Zugegriffen: 14. Dez. 2022.

[CR4] Bayer J, Landesanstalt für Medien NRW (2020). Disinformation and its certainly regulatory approaches. Was ist Desinformation? Betrachtungen aus sechs wissenschaftlichen Perspektiven.

[CR71] Benkler, Y. (2018). Are the Russians coming? In Y. Benkler, R. Faris, & H. Roberts (Hrsg.), *Network propaganda: manipulation, disinformation and radicalization in American politics* (S. 235–268). Oxford: Oxford University Press. 10.1093/oso/9780190923624.001.0001

[CR72] Benkler, Y. (2019). Cautionary notes on disinformation and the origins of distrust. Social Science Research Council. https://mediawell.ssrc.org/expert-reflections/cautionary-notes-on-disinformation-benkler/. Zugegriffen: 19. Dez. 2022.

[CR7] BMI – Bundesministerium des Innern und für Heimat (2022). Top-Thema „Desinformation als Hybride Bedrohung“. https://www.bmi.bund.de/SharedDocs/topthemen/DE/topthema-desinformation/artikel-desinformation-hybride-bedrohung.html. Zugegriffen: 30. Nov. 2022.

[CR9] Bond, S. (2022, 21. Mai). She joined DHS to fight disinformation. She says she was halted by… disinformation. NPR. https://www.npr.org/2022/05/21/1100438703/dhs-disinformation-board-nina-jankowicz. Zugegriffen: 14. Dez. 2022.

[CR10] BPA – Bundespresseamt (2022). Recht auf Information. https://www.bundesregierung.de/breg-de/bundesregierung/bundeskanzleramt/bundespresseamt/recht-auf-information-460940. Zugegriffen: 30. Nov. 2022.

[CR11] Bundesregierung (2022). Begriffserklärung: Was ist Desinformation? https://www.bundesregierung.de/breg-de/themen/umgang-mit-desinformation/was-ist-desinformation-1875148. Zugegriffen: 30. Nov. 2022.

[CR12] Bundestag (2022). „Echokammern“ und „Filterblasen“ in digitalen Medien. https://www.bundestag.de/resource/blob/898208/396d70db93fbc68bca40726b4d5308db/WD-10-007-22-pdf-data.pdf. Zugegriffen: 1. Dez. 2022.

[CR13] Bussemer T (2008). Propaganda. Konzepte und Theorien.

[CR14] DHS (2022). Following HSAC recommendation, DHS terminates disinformation governance board. https://www.dhs.gov/news/2022/08/24/following-hsac-recommendation-dhs-terminates-disinformation-governance-board. Zugegriffen: 14. Dez. 2022.

[CR15] Europäische Kommission (2018). Aktionsplan gegen Desinformation. https://www.eeas.europa.eu/sites/default/files/aktionsplan_gegen_desinformation.pdf. Zugegriffen: 1. Dez. 2022.

[CR16] Europäisches Parlament (2016, 4. Juli). Nato strategic communications – An evolving battle of narratives. https://www.europarl.europa.eu/thinktank/en/document/EPRS_BRI(2016)586600. Zugegriffen: 14. Dez. 2022.

[CR17] EUvsDisinfo – East StratCom Task Force (2021, 9. März). Vilifying Germany, wooing Germany. https://euvsdisinfo.eu/vilifying-germany-wooing-germany2/. Zugegriffen: 1. Dez. 2022.

[CR18] Fridman O (2020). ‘Information War’ as the Russian conceptualisation of strategic communications. The RUSI Journal.

[CR19] Fuhrberg R, Kocks K, Knorre S, Kocks JN (2020). Verhaltensökonomie in der Verwaltungskommunikation – Der Staat als Entscheidungsarchitekt. Öffentliche Verwaltung – Verwaltung in der Öffentlichkeit.

[CR20] Gigerenzer G (2018). The bias bias in behavioral economics. Review of Behavioral Economics.

[CR21] Graf R (2018). Verhaltenssteuerung jenseits von Markt und Moral. Die umweltpolitische Regulierungsdiskussion in der Bundesrepublik Deutschland und den USA im letzten Drittel des 20. Jahrhunderts. Vierteljahreshefte für Zeitgeschichte.

[CR22] Gruhl S, Bell C, Hark F, Huber P, König L, Zowislo-Grünewald N, Wörmer N (2021). Hybride Bedrohungen im Informationsumfeld. Herausforderungen für die deutsche Sicherheitspolitik im 21. Jahrhundert. Kommunikation, Resilienz und Sicherheit.

[CR23] Hölig S, Behre J, Schulz W (2022). Reuters Institute Digital News Report 2022: Ergebnisse für Deutschland.

[CR24] Ingram HJ (2020). The strategic logic of state and non-state malign ‘influence activities’. Polarising populations, exploiting the democratic recession. The RUSI Journal.

[CR36] Jankowicz N (2020). How to lose the information war. Russia, fake news and the future of conflict.

[CR26] Janzen J (2019). What if the pen is a sword? Communicating in a chaotic, sensational, and weaponized information environment. Canadian Military Journal.

[CR25] Jaursch J, Sängerlaub A, LfM NRW (2020). Desinformation als Arbeitsfeld der Medienaufsicht. Was ist Desinformation? Betrachtungen aus sechs wissenschaftlichen Perspektiven.

[CR27] Jordan J (2020). International competition below the threshold of war. Journal of Strategic Security.

[CR28] Kahneman D (2011). Schnelles Denken, langsames Denken.

[CR29] Karpf, D. (2019, 10. Dez.). On digital disinformation and democratic myths. Social Science Research Council. https://mediawell.ssrc.org/expert-reflections/on-digital-disinformation-and-democratic-myths/. Zugegriffen: 7. Dez. 2022.

[CR30] Karpf D, Bennett WL, Livingston S (2020). How disinformation turned dangerous. The disinformation age.

[CR69] Knappenschneider H, Feige J, Zowislo-Grünewald N, Wörmer N (2021). Counter influence campaigns as integral part of policy planning for resilience. Kommunikation, Resilienz und Sicherheit.

[CR32] Kocks K, Knorre S, Kocks JN (2020). Öffentliche Verwaltung – Verwaltung in der Öffentlichkeit.

[CR31] Kraft I (2018). Hybrider Krieg – zu Konjunktur, Funktion und Dynamik eines Konzepts. Zeitschrift für Außen- und Sicherheitspolitik.

[CR70] KSTA – Kölner Stadt-Anzeiger (2022, 8. Nov.). Krieg gegen die Ukraine: Mehr Deutsche glauben russischer Propaganda. https://www.ksta.de/politik/ukraine-krieg-mehr-deutsche-glauben-russischer-propaganda-369161?cb=1671444104194. Zugegriffen: 18. Dez. 2022.

[CR33] Lamberty, P., Heuer, C., & Holnburger, J. (2022). Belastungsprobe für die Demokratie: Pro-russische Verschwörungserzählungen und Glaube an Desinformation in der Gesellschaft. CeMAS. https://cemas.io/publikationen/belastungsprobe-fuer-die-demokratie/2022-11-02_ResearchPaperUkraineKrieg.pdf.

[CR34] Lamoureux, M. (2021, 30. Sep.). Conspiracy theorists are salivating over a canadian military psy-op report. Vice. https://www.vice.com/en/article/k78mqw/conspiracy-theorists-are-salivating-over-a-canadian-military-psy-op-report. Zugegriffen: 14. Dez. 2022.

[CR37] Mast T (2020). Staatsinformationsqualität. De- und Rekonstruktion des verfassungsgerichtlichen Leitbilds öffentlicher und staatlicher Informationstätigkeit und der entsprechenden Gebote.

[CR38] Mau S (2022). Kamel oder Dromedar? Zur Diagnose der gesellschaftlichen Polarisierung. Merkur.

[CR39] MDR (2020, 11. Nov.). Russische Propaganda wirkt – vor allem im Osten. https://www.mdr.de/nachrichten/deutschland/gesellschaft/recap-russische-propaganda-ostdeutschland-100.html. Zugegriffen: 14. Dez. 2022.

[CR40] Meier, C., & Hoffmann, C. (2022, 14. Sep.). Politische Eliten müssen Kritik aushalten und ernst nehmen. Welt. https://www.welt.de/kultur/medien/plus240955457/Medienwissenschaftler-Politische-Eliten-muessen-Kritik-aushalten.html. Zugegriffen: 7. Dez. 2022.

[CR41] Meister, S. (2022, 20. Mai). Russische Propaganda. Als wäre es die Wahrheit. Die Zeit. https://www.zeit.de/politik/ausland/2022-05/russische-propaganda-ukraine-krieg-westen-manipulation. Zugegriffen: 7. Dez. 2022.

[CR73] Möller J, Hameleers M, Medien NRW (2020). Different types of disinformation, its political consequences and treatment recommendations for media policy and practice. Was ist Desinformation? Betrachtungen aus sechs wissenschaftlichen Perspektiven.

[CR43] Nimmo, B. (2018). Question that: RT’s military mission. Assessing Russia Today’s role as an “information weapon”. https://medium.com/dfrlab/question-that-rts-military-mission-4c4bd9f72c88 . Zugegriffen: 7. Dez. 2022.

[CR44] Nordheim, G. von (2020, 16. Sep.). Das große Rauschen. Journalismus zwischen Propaganda und Polarisierung. Medium. https://medium.com/@gvnordheim/das-gro%C3%9Fe-rauschen-e67895a19ad9. Zugegriffen: 8. Dez. 2022.

[CR45] Nyhan B (2020). Facts and Myths about Misperceptions. Journal of Economic Perspectives.

[CR46] Pugliese, D. (2021, 27. Sep.). Military leaders saw pandemic as unique opportunity to test propaganda techniques on Canadians, Forces report says. Ottawa Citizen. https://ottawacitizen.com/news/national/defence-watch/military-leaders-saw-pandemic-as-unique-opportunity-to-test-propaganda-techniques-on-canadians-forces-report-says. Zugegriffen: 14. Dez. 2022.

[CR47] Reimann, P. (2022, 7. Okt.). Der unsichtbare Feind – Wer beeinflusst Soldaten? Tagesspiegel. https://background.tagesspiegel.de/cybersecurity/der-unsichtbare-feind-wer-beeinflusst-soldaten. Zugegriffen: 7. Dez. 2022.

[CR48] Ross Arguedas, A., Robertson, C. T., Fletcher, R., & Nielsen, R. K. (2022). Echo chambers, filter bubbles, and polarisation: A literature review. Reuters Institute for the Study of Journalism. https://reutersinstitute.politics.ox.ac.uk/sites/default/files/2022-01/Echo_Chambers_Filter_Bubbles_and_Polarisation_A_Literature_Review.pdf.

[CR49] Rühle, M., & Roberts, C. (2021, 19. März). Enlarging NATO’s toolbox to counter hybrid threats. NATO Review. https://www.nato.int/docu/review/articles/2021/03/19/enlarging-natos-toolbox-to-counter-hybrid-threats/index.html. Zugegriffen: 7. Dez. 2022.

[CR50] Sächsische Zeitung (2022, 2. Nov.). Pro-russische Propaganda überzeugt immer mehr Ostdeutsche. https://www.saechsische.de/russland/russland-propaganda-ostdeutschland-krieg-putin-ukraine-5776714.html. Zugegriffen: 14. Dez. 2022.

[CR51] SCCOE – NATO Strategic Communications Center of Excellence (2020). About Strategic Communications. https://stratcomcoe.org/about_us/about-strategic-communications/1. Zugegriffen: 7. Dez. 2022.

[CR52] Schaurer F, Ruff-Stahl H-J (2016). Hybride Bedrohungen. Sicherheitspolitik in der Grauzone. Aus Politik und Zeitgeschichte.

[CR53] Schmid J, Böckenförde S, Gareis S (2021). Herausforderungen hybrider Kriegführung. Deutsche Sicherheitspolitik. Herausforderungen, Akteure und Prozesse.

[CR54] Silver, L. (2021, 5. Mai). Ideological divisions over cultural issues are far wider in the U.S. than in the UK, France and Germany. Pew Research Center. https://pewrsr.ch/3b2IZ3J. Zugegriffen: 8. Dez. 2022.

[CR55] Steindl C, Jonas E, Sittenthaler S, Traut-Mattausch E, Greenberg J (2015). Understanding Psychological Reactance. New Developments and Findings. Zeitschrift für Psychologie.

[CR56] Teetz A, Möllers H, Jacobs J (2019). Rechenschaft vor ihresgleichen. Der „Staatsbürger in Uniform“ als kommunikatives Leitbild in der digitalen Mediengesellschaft. Bundeswehr und Medien. Ereignisse - Handlungsmuster - Mechanismen in jüngster Geschichte und heute.

[CR57] Thaler R, Sunstein C (2011). Nudge: Wie man kluge Entscheidungen anstößt.

[CR58] UK Government (2015). Government Communications Plan 2015/16. https://communication-plan.gcs.civilservice.gov.uk/wp-content/uploads/2019/04/GCS-Government-Communications-Plan-1516-1.pdf. Zugegriffen: 8. Dez. 2022.

[CR59] UK Government (2018). Strategic communication: a behavioural approach. https://gcs.civilservice.gov.uk/wp-content/uploads/2020/03/Strategic-Communications-a-behavioural-approach.pdf. Zugegriffen: 8. Dez. 2022.

[CR60] UK Government (2020). Guide to campaign planning: OASIS. https://gcs.civilservice.gov.uk/guidance/marketing/delivering-government-campaigns/guide-to-campaign-planning-oasis/. Zugegriffen: 8. Dez. 2022.

[CR61] UK MoD (2019). Joint Doctrine Note 2/19. Defence strategic communication: an approach to formulating and executing strategy. https://assets.publishing.service.gov.uk/government/uploads/system/uploads/attachment_data/file/804319/20190523-dcdc_doctrine_uk_Defence_Stratrategic_Communication_jdn_2_19.pdf. Zugegriffen: 8. Dez. 2022.

[CR62] UK MoD (2021). The strategic command strategy. Policy paper. https://www.gov.uk/government/publications/the-strategic-command-strategy/the-strategic-command-strategy. Zugegriffen: 14. Dez. 2022.

[CR63] Westheuser L (2022). This is not America: Politische Polarisierung in Deutschland als Schimäre. Forschungsjournal Soziale Bewegungen.

[CR64] Wigell, M., Mikkola, H., & Juntunen, T. (2021). Best practices in the whole-of-society approach in countering hybrid threats. European Parliament. https://www.europarl.europa.eu/RegData/etudes/STUD/2021/653632/EXPO_STU(2021)653632_EN.pdf. Zugegriffen: 8. Dez. 2022.

[CR65] Zeit Online (2022, 3. Nov.). Immer mehr Deutsche stimmen laut Studie russischer Propaganda zu. https://www.zeit.de/politik/deutschland/2022-11/cemas-studie-russland-propaganda. Zugegriffen: 14. Dez. 2022.

[CR66] Zerfass, A. (2015). Was bringt das alles? Wertschöpfung durch Kommunikation. Communication Insights. https://www.econstor.eu/bitstream/10419/178663/1/communication-insights-1.pdf. Zugegriffen: 8. Dez. 2022.

[CR68] Zowislo-Grünewald N, Hajduk J, Zowislo-Grünewald N, Wörmer N (2021). Strategisches Kommunikationsmanagement als Domain der wehrhaften Demokratie. Kommunikation, Resilienz und Sicherheit.

[CR67] Zowislo-Grünewald N, Wörmer N (2021). Kommunikation, Resilienz und Sicherheit.

